# Self-Reported Stickiness of Mind-Wandering Affects Task Performance

**DOI:** 10.3389/fpsyg.2016.00732

**Published:** 2016-05-18

**Authors:** Marieke K. van Vugt, Nico Broers

**Affiliations:** Institute of Artificial Intelligence and Cognitive Engineering, University of GroningenGroningen, Netherlands

**Keywords:** mind-wandering, current concerns, distraction, SART, sustained attention, goals

## Abstract

When asked to perform a certain task, we typically spend a decent amount of time thinking thoughts unrelated to that task–a phenomenon referred to as “mind-wandering.” It is thought that this mind-wandering is driven at least in part by our unfinished goals and concerns. Previous studies have shown that just after presenting a participant with their own concerns, their reports of task-unrelated thinking increased somewhat. However, effects of these concerns on task performance were somewhat inconsistent. In this study we take the opposite approach, and examine whether task performance depends on the self-reported thought content. Specifically, a particularly intriguing aspect of mind-wandering that has hitherto received little attention is the difficulty of disengaging from it, in other words, the “stickiness” of the thoughts. While presenting participants with their own concerns was not associated with clear effects on task performance, we showed that the reports of off-task thinking and variability of response times increased with the amount of self-reported stickiness of thoughts. This suggests that the stickiness of mind-wandering is a relevant variable, and participants are able to meaningfully report on it.

## Introduction

While most of experimental psychology is based on the idea of manipulating behavior with different task conditions, a small body of research looks at what happens when participants do not perform the task but instead become distracted (Antrobus et al., 1970; Klinger, 1990; Smallwood and Schooler, 2014). In that literature, distraction is usually defined as task-unrelated thinking, a variant of mind-wandering. Because by definition task-unrelated thinking is not a task, it is measured indirectly with a variety of methods including response time variability (which is increased when task performance is contaminated with task-unrelated processes; Bastian and Sackur, 2013), event-related potentials (which are typically reduced relative to task-related stimuli when someone is mind-wandering; Smallwood et al., 2008; Kam et al., 2011; Macdonald et al., 2011) and pupil size (which responds less to presented stimuli when someone is mind-wandering; Smallwood et al., 2011; Mittner et al., 2014). However, the most frequently used method to examine task-unrelated thinking involves “thought probes,” in which the task is intermixed with questions such as “were you just now on-task, or thinking about something else?” (Smallwood et al., 2004). Based on these responses, task performance can be compared between the periods just prior to reports of being off-task and periods just prior to reports of being on-task. Usually, off-task reports are associated with worse task performance in terms of response time or accuracy (McVay and Kane, 2009), and especially response time variability (Bastian and Sackur, 2013).

There are many different theories about what triggers task-unrelated thinking (see Smallwood and Schooler, 2014 for a review). These theories range from a failure of executive control (McVay and Kane, 2010) to decoupling from the environment such that external stimuli become less important (Smallwood et al., 2011) to preoccupation with current concerns (Klinger, 1966, 1977, 2013). Each of these theories has been developed in the context of a specific (set of) tasks. It is likely that not one theory of mind-wandering is correct, but that different task situations lead to the prevalence of mind-wandering triggered by different factors.

The “current concerns” theory of mind-wandering—which states that goals or problems that currently occupy a person tend to intrude on thinking processes—has received less attention in recent times. One reason for this may be that it is difficult to manipulate and control current concerns in a task. Nevertheless, McVay and Kane (2013) developed a task that could manipulate concerns, and demonstrated that task-unrelated thinking increased when participants were presented with stimuli priming their on-going goals and concerns. Just after presenting such concerns, participants showed an increased tendency to report being off-task relative to non-self-related goals. This effect occurred despite the fact that participants were completely unaware of the fact that any concerns were inserted in the task, suggesting that concern-related thinking is largely automatic. Concerns were also associated with a slight decrease in task accuracy. While McVay and Kane's task was a variant of the sustained attention to response task (Robertson et al., 1997), it has also been shown that reminding participants of unfulfilled goals increased intrusive thoughts during a reading task (Masicampo and Baumeister, 2011).

It is unclear why the behavioral effects of reminding people of their concerns is relatively weak and inconsistent if concerns are such an important driving factor of mind-wandering. One potential explanation is that the amount and type of mind-wandering triggered by concerns is not precisely time-locked to the presentation of the concerns (but the data analyses are). If that is the case, declines in task performance may only occur at the moment that the concern becomes so engrossing that task priorities are more or less forgotten, and this could occur at a random moment after concerns have been presented. It has been suggested that mind-wandering related to unattained goals is particularly rigid, inflexible, and narrowly-focused (Marchetti et al., 2016). Joormann et al. (2011) introduced the idea of “sticky” modes of thinking in which people “have difficulty keeping irrelevant negative material from entering working memory” and manipulating it. Sticky modes of thinking consist of trains of thought that are difficult to disengage from (van Vugt et al., 2012). This tendency is also associated with a habit of rumination, i.e., recurrent, uncontrolled and habitual negative thought (Nolen-Hoeksema et al., 2008). While rumination is restricted to clinical samples, Verplanken et al. (2007) developed a questionnaire to assess the tendency to engage in habitual negative thinking that is applicable to more general populations. This questionnaire uses a participant's own metacognitive insight to measure the degree to which negative self-related thinking occurs frequently, is initiated without awareness and is difficult to control. Verplanken and colleagues observed that scoring high on this questionnaire was associated with lower self-esteem, an increase in the speed of processing of negative self-related stimuli, and increased symptoms of anxiety and depression. In addition, the score on this questionnaire was associated with implicit and explicit measures of body dissatisfaction (Verplanken and Tangelder, 2011), which is another manifestation of a concern people may have.

Together, these studies suggest that there is a “sticky” dimension of off-task thinking, which reflects its tendency to be repetitive, uncontrolled, habitual, and difficult to disengage from. The material that tends to give rise to uncontrolled and habitual thinking is often a concern or goal that is unfinished. These goals can range from positively-valenced planning for the future to negatively-valenced worry about whether one can even solve these concerns. Worries related to the self could also fall in this category, because negative self-related thinking often revolves around the abstract goals of being “good enough” and performing well. With this definition of sticky thinking, it may well be the case that in the current concerns task, such sticky thinking is primed. Such thinking can increase and decrease in strength over the course of the experiment, and may occur largely under the conscious surface (Dijksterhuis and Nordgren, 2006). The moment a participant latches onto such cues, a sticky thought process may be set in motion. However, this may not always occur reliably time-locked to the appearance of words that reflect these concerns. If this is the case, that could explain why in a current concerns mind-wandering task, there can be little difference in task performance between the concern conditions, as McVay and Kane observed. It also suggests that when relying on participant's introspection or meta-cognitive insight instead of the task conditions to find out when sticky thinking occurs, this could in fact reveal decreases in task performance. Specifically, such momentary increases in stickiness should be associated in particular with increases in response time variability and off-task thinking.

Another dimension of off-task thinking that has been distinguished is its temporal focus. Stawarczyk et al. (2011) demonstrated that after asking participants to write about their current goals, participants exhibited more future-related thoughts in a sustained-attention to response task. Ruby et al. (2013) demonstrated that thoughts that were past-related predicted future negative mood, while thoughts that were future-related predicted future positive mood on a simple choice response task. Plimpton et al. (2015) found in a vigilance task that dysphoric participants reported less thinking about the future, and more about the past when presented with thought probes. In addition, positive words presented on the screen triggered more future-related thoughts relative to negative words. While in general future-related thinking is posited to be adaptive and more prevalent than past-related thinking, other studies have shown that when given the option, participants report mostly an atemporal focus (Jackson et al., 2013). Yet, none of these studies directly assessed the effect of temporal focus on task performance. We therefore decided to explore the temporal dimension of task-unrelated thinking in addition to its stickiness dimension.

To examine the sticky and temporal dimensions of off-task thinking and its effect on behavior, we used the current concerns paradigm introduced by McVay and Kane (2013). This paradigm consisted of a pre-experiment questionnaire to collect a participant's concerns, and an actual SART task in which these concerns were embedded. Critically, we added thought probe questions to assess the degree of stickiness and temporal focus to test whether it is the task condition that leads to declines in performance, or rather the mode of the thinking.

## Materials and methods

### Participants

The 26 participants (12 female, age mean: 21.7, range: 19–29) were Dutch native speakers from the city of Groningen community and were compensated with a monetary reward of €17 for their participation. The experimental procedures were in agreement with the Declaration of Helsinki and all participants signed an Informed Consent form. Participants were excluded if their go-trial errors exceeded 50% of all trials, suggesting random guessing. None of the participants exhibited such an error rate. One participant was excluded from further analysis because she/he aborted the experiment half-way.

### Materials

#### Questionnaire session

Participants were requested to fill out three questionnaires online prior to the experiment. The first two of these questionnaires served as fillers to distract from the purpose of the experiment. Participants filled in the Cognitive Failures Questionnaire (McVay and Kane, 2009, 2013), the Habit Index of Negative Thinking (Verplanken et al., 2007) and the Personal Concerns Inventory (PCI; adapted from Cox and Klinger, 2004). In the PCI, participants wrote a short statement expressing a current concern/goal in their lives to each of nine categories: (1) Home and Household Matters, (2) Employment and Finances, (3) Partner, Family and Relatives, (4) Friends and Acquaintances, (5) Spiritual Matters, (6) Self Changes, (7) Education and Training, (8) Health and Medical Matters, (9) Hobbies, Pastimes and Recreation. Items of the PCI that were judged to be inappropriate for our research setting were dropped (e.g., Substance Use; Love, Sex and Intimacy). Participants rated the importance of each concern on a Likert-Scale ranging from 1 (not at all important) to 10 (the most important concern/goal in my life right now) and provided a temporal estimate when the goal/concern would be accomplished/resolved. Participants were encouraged to focus on concerns that were issues on the time scale of at most 2 years.

#### Experimental session

Materials were 360 English-to-Dutch translated words, drawn quasi-randomly from the Battig-Montague word pool (Battig and Montague, 1969). This word database consists of 56 categories, split into verbal taxonomies covering geography (e.g., countries), nature (e.g., flower), different animals (e.g., bird, fish) and a wide range of other categories used in common language. Given that the word pool originated from American culture, certain words were translated to fit Dutch culture. For instance, “John” from the category “names” was changed to the Dutch name “Daan” or “South Dakota” from the category “States” was changed to the Dutch province “Drenthe.”

### Task

**Figure 1:**
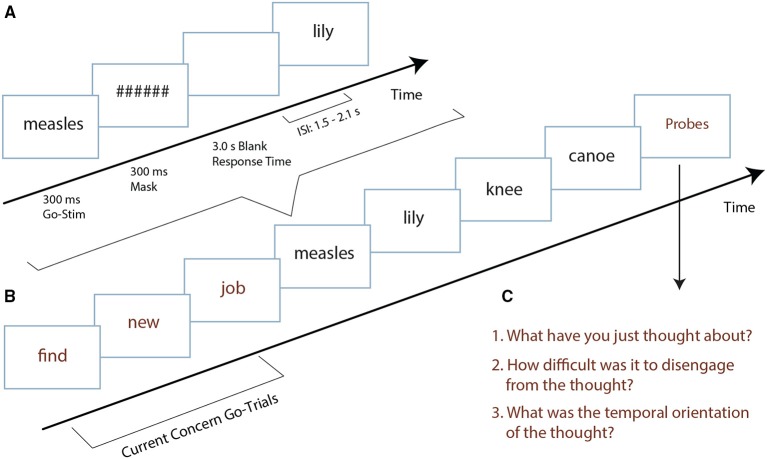
**(A)** Temporal structure of an individual trial. **(B)** Trial structure. In this example there are four trials in-between the current concern trials and the thought probes. **(C)** Thought probe questions. The first question asks about the content, the second asks about the stickiness of the content, the third asks about the temporal orientation of that content (stimuli were translated from Dutch to English for display purposes).

Two of the most significant concerns per participant were extracted from the Personal Concern Inventory and translated into three-word structures (e.g., find - new - job), with each word serving as a stimulus in the SART (see Figure 1B) in three consecutive trials. If two concerns were rated equally important, the more imminent concern was selected. We also extracted two control statements from participants from the pilot study that were relatively idosyncratic. These control statements were used for the subsequent participants as control statements.

We adopted the SART task from McVay and Kane (2013) but implemented two changes: (1) We reduced the number of trials by a third to reduce the time of the session from 90 to 60 min. In a pilot experiment, participants complained about the duration and this complaint was mirrored in exceptionally short response times in the last 30 min. (2) We implemented more trials between the current concern triplets and the thought probes. While McVay and Kane (2013) used one, three and five trials, we increased this to either four or five, counterbalanced across the experiment (see Figure 1B). We adapted this interval because we were interested in response time variability after the current concern triplets had been shown and needed a representative amount of trials to be able to compute that number. In eight blocks of 90 trials respectively, each block contained two repetitions of the same 45 trials, in which one current concern and one control concern triplet were included (which were the only things that differed between the two repetitions). The two current and control concerns alternated across blocks and were counterbalanced across the experiment. After each concern triplet, four/five trials preceded our set of probe questions (see Figures 1B,C). Per block, one additional control probe was randomly inserted, not following a specific concern. This probe will be used as a non-concern control condition. Each block of 90 trials therefore contained two current concern and two other concern trial triplets and four associated thought probes (consisting of three questions each), as well as two control thought probes inserted at random points in the task. Of the 90 trials in each block, 10 were no-go trials (approximately 11% of the trials).

### Procedure

Participants completed the questionnaires in an online survey (see section “Materials”), a week before the experimental session. In the experimental session, we adopted the perceptual version of the SART from McVay and Kane (2009, 2013). Participants completed a prolonged go/no-go task and were instructed to press the space bar as quickly as possible when they see a word in lower case (e.g., “lily”) and to withhold a response when a word is printed in upper case (e.g., “BANANA”). The words were presented and masked for 0.3 s each and participants had 3.6 s in total to respond (Figure 1A). Time between trials was randomly picked from a uniform distribution ranging from 1.5 to 2.1 s. Four or five trials after the concern structures, participants were shown “thought probes”—questions probing their subjective experience. Specifically, the three questions interrogated the content, stickiness level and temporal orientation of the thoughts just preceding the probe (see Figure 1C). The first question replicated McVay and Kane (2013), and pertains to the nature of the participant's thoughts just preceding the question, asking whether they thought about (1) the task, (2) task performance, (3) everyday life, (4) current state of being, (5) personal concerns, (6) day dreams, (7) other. We subsequently classified responses larger than 2 as “off-task” and responses 1 and 2 as “on-task.” The results do not change qualitatively when taking only 1 as an index of on-task. The other two questions had not been used in previous research on the current concerns task. The second question was derived from the Habit Index of Negative Thinking (Verplanken et al., 2007), and asked about how difficult it was to disengage from the previous thought and participants indicated on a Likert-Scale ranging from 1 (very difficult) to 5 (very easy) the stickiness level of the thought. The coding of this Likert scale was reversed for the analyses for ease of interpretation. The third question was similar to one used in previous mind-wandering experiments (Smallwood et al., 2011; Ruby et al., 2013), asking about the temporal orientation of the thought, i.e., whether it was related to (1) the past, (2) the present or (3) the future. After four blocks, participants were given the opportunity to take a break. The experiment ended with a debriefing, in which the participant was first asked whether they noticed anything special about the words, and then was explained the actual purpose of the experiment. None of our participants indicated noticing their concerns were embedded in the stimulus stream.

### Apparatus

The questionnaire was created, distributed and controlled with Google Forms. Stimuli were presented and the registration of responses was controlled with PsychoPy 1.83 (Peirce, 2007). The experiment ran on a Mac Mini with a 2.4 GHz Intel Core 2 Duo processor and a 21-inch LCD monitor, with a 1920 × 1080 resolution and a 60 Hz refresh rate.

### Data analysis

We computed the average response time, accuracy, and coefficient of variance of response time on the 8 trials preceding each thought probe. We compared these values between the different task conditions and different levels of responses on the thought probes by means of linear mixed effects models (Bates et al., 2015). Linear mixed effects models were used because they are robust to missing or unbalanced data. The effect of an experimental factor was tested by comparing the models with and without that factor by means of a chi-squared test. When the model fits improved significantly according to the chi-square statistic, the factor was judged significant. When the factor was significant, the reliability of individual contrasts was determined by means of a *post-hoc t*-test on the relevant regression coefficients (using the R package lsmeans).

## Results

**Figure 2:**
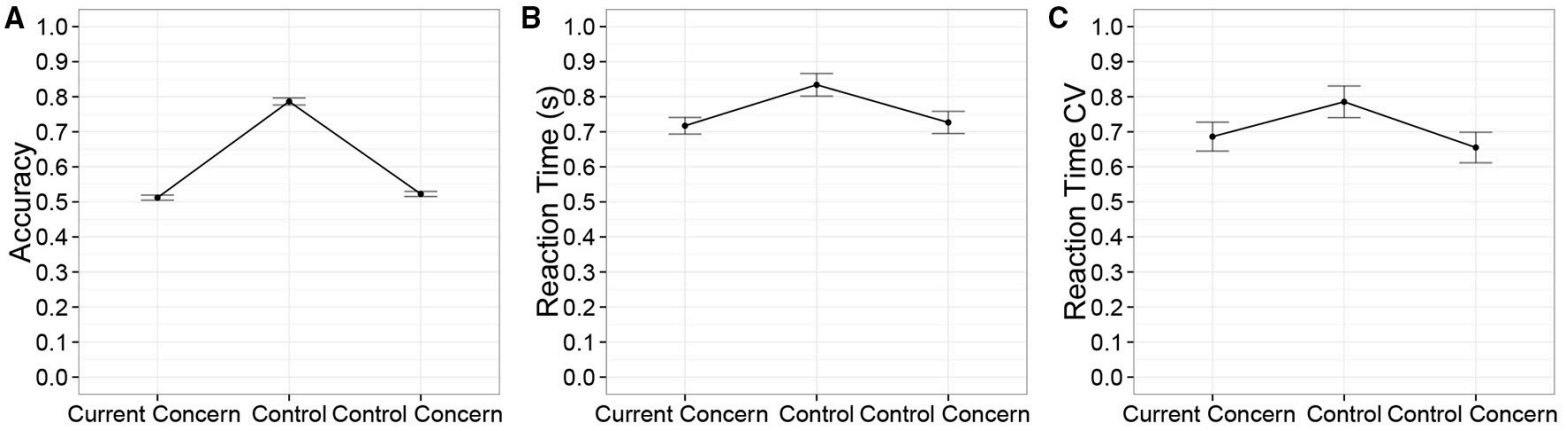
**The effect of current concern condition on (A) accuracy, (B) response time, and (C) the coefficient of variation (CV) of response time**. Error bars reflect standard errors.

We first checked how inserting current concerns in the stimulus stream affected task performance and off-task thinking. We found that accuracy was higher in the non-concern control condition than in either of the two concern conditions [Figure 2A, $\chi_{\left(2\right)}^{2}=452.7$, *p* < 0.001]. Just like McVay and Kane did not observe a consistent difference in accuracy between control concerns and personal concerns, we did not either [*post-hoc t*_(475)_ = −0.8, *n*.*s*.]. They did not have the non-concern control condition, for which we observed a clear increase in accuracy.

Unlike McVay and Kane, we also report on response times. Response times were shorter in the concern conditions relative to the control condition [Figure 2B, $\chi_{\left(2\right)}^{2}=9.0$, *p* < 0.05]. The coefficient of variation of response time (RTCV) has previously been thought to reflect mind-wandering. We observed that in contrast to our expectation of more mind-wandering after priming the participant with concerns, the coefficient of variation of response time (RTCV) showed a trend toward being larger in the control condition relative to the two concern conditions [Figure 2C, $\chi_{\left(2\right)}^{2}=5.3$, *p* = 0.07].

**Figure 3:**
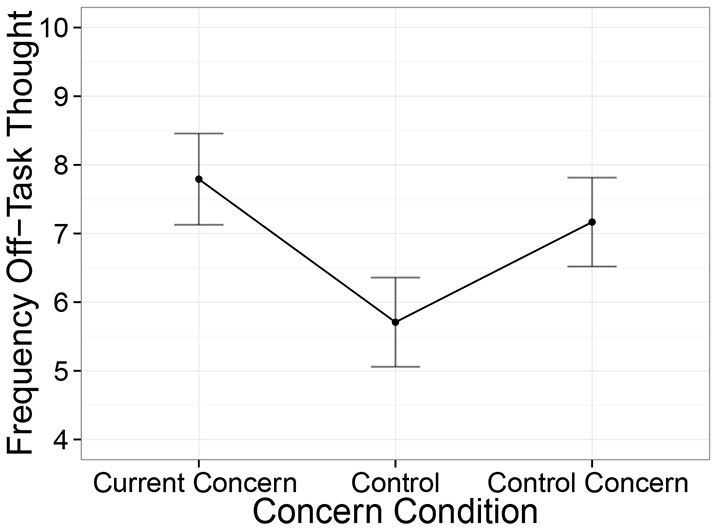
**Frequency of off-task thinking by current concern condition**. Number of off-task thinking reports is increased in the current concerns condition relative to the control condition. Error bars reflect standard errors.

If current concerns increase the amount of off-task thinking, then off-task thinking should be increased just after current concern stimuli, and this should be—as McVay and Kane observed–larger for own concerns relative to other concerns. Figure 3 shows that the reported frequency of off-task thinking differed between the conditions [$\chi_{\left(2\right)}^{2}=12.5$, *p* < 0.01). Specifically, off-task thinking was more frequent in the current concern condition than in the non-concern control condition [*t*_(46)_ = 3.6, *p* < 0.001], but it did not differ between the participant's own and other's concerns [*t*_(46)_ = 1.1, *n*.*s*.].

**Figure 4:**
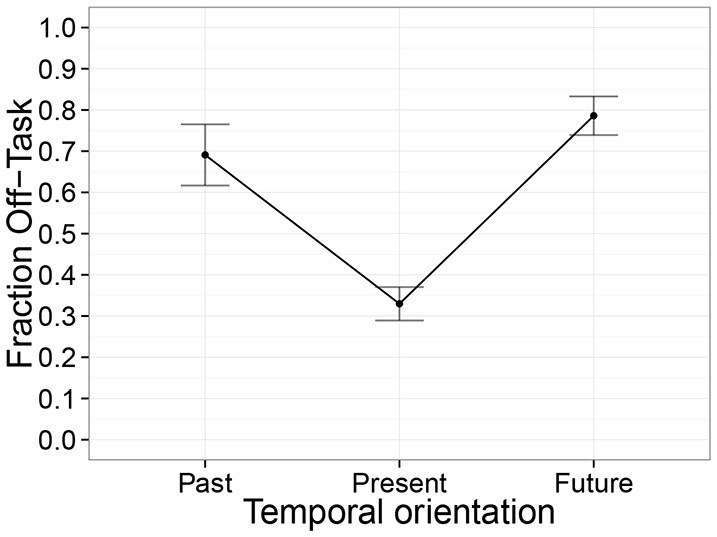
**Fraction of off-task thinking as a function of the temporal orientation of these thoughts**. Error bars reflect standard errors.

Since the effects of the concern conditions on behavior were relatively moderate, we then investigated whether temporal orientation was related to the frequency of off-task thinking. Indeed, we observed that the frequency of off-task thinking differed significantly between temporal orientations [Figure 4, $\chi_{\left(2\right)}^{2}=34.3$, *p* < 0.001]. Specifically, the “present” responses were associated with significantly less frequent off-task thinking than the “past” responses [*t*_(46.7)_ = 4.8, *p* < 0.001] and “future" responses [*t*_(46.7)_ = 6.3, *p* < 0.001].

**Figure 5:**
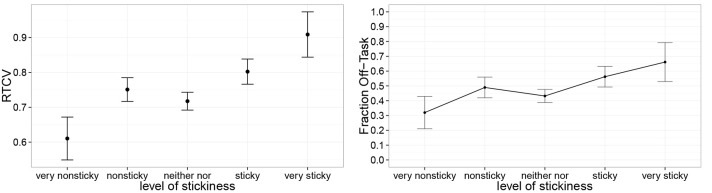
**Off-task thinking (right) and variability in response time (left) increases with self-reported thought stickiness**. Error bars reflect standard errors.

We have hypothesized that the sticky nature of the content of thinking is what makes task performance decline. To test this hypothesis, we asked whether participants reported more frequent off-task thinking when they responded that it was difficult to disengage from the thought (our operationalization of “sticky thinking”). We found that the frequency of off-task thinking decreased as the stickiness of the thought decreased [Figure 5, $\chi_{\left(1\right)}^{2}=6.4$, *p* < 0.05].

Finally, we asked whether this self-reported stickiness was also associated with response time variability. It has been suggested the variability in response time is a sensitive measure of mind-wandering (Bastian and Sackur, 2013). Indeed, Figure 5 shows that as stickiness increased, the coefficient of variation of response time increased [$\chi_{\left(1\right)}^{2}=4.1$, *p* < 0.05].

## Discussion

We showed–just like McVay and Kane (2013)–that current concerns had only small effects on task performance. However, when examining the data from the perspective of the contents of their thought processes, we found clear effects on the probability of off-task thinking and variability in response times. Specifically, in line with ideas from the field of clinical psychology, the more difficult it is to disengage from the thinking, the more likely the person is off-task, and the more variable their response times. We also showed that future and past thoughts were more often off-task than present-related thoughts.

Together, this suggests that current concerns do not reliably induce off-task thinking, but when off-task thinking does occur it seems to affect task performance. To consider why inserting concerns does not reliably affect task performance (at least not time-locked to the concern probes) it is instructive to discuss current theories about how concerns could affect task performance. Hiatt and Trafton (2015) created a computational cognitive model of the current concerns task based on the idea that mind-wandering kicks off when there is a natural break in the task. Such a break occurs when one is not actively reasoning toward a particular goal, and at the same time there is an active thought in working memory that can set off a train of mind-wandering. In their model, the increase in response time variability would be explained by an increase in the frequency of thoughts “popping” into working memory, which disrupts performance of the main task. Their model does not consider a dimension of thought stickiness because the model simply halts when it is distracted.

To be able to explain the effect of thought stickiness, the mind-wandering process itself needs to be explicitly modeled as a sequence of episodic retrievals, as we have done in another computational model (van Vugt et al., 2015). In that model, increased stickiness could be modeled as an increased mental distance between the sticky thought and thoughts related to task goals in episodic memory. This increased distance makes it difficult to return to the task, which relies on retrieving the task goal. In contrast, continuing to mind-wander is fairly easy. Although the appearance of a concern word may increase the probability of it being retrieved, thereby leading one further away from task goals, this mind-wandering does not need to be exactly time-locked to the appearance of these concerns. Indeed, off-task thinking was associated with a significantly lower task accuracy than on-task thinking [$\chi_{\left(1\right)}^{2}$ = 6.2, *p* < 0.05], although it did not affect RT and its variability. In addition, it may well be the case that only concerns that are associated with worries of the participant lead to task disruption. Since concerns defined in our study could both be goals that have a relatively positive affective value and concerns that are associated with worry, this mixture of concern types may also explain the inconsistent effects.

One puzzling finding in our study of the current concerns task was that there appeared to be a change in speed-accuracy trade-off across conditions, such that current concerns were associated with a decrease in accuracy, but also a decrease in response time and its variability. Such changes in speed-accuracy have also been reported in previous studies using the SART. For example, Seli et al. (2012) demonstrated that when participants were explicitly cautioned to respond slowly, then accuracy increased together with response time. Nevertheless, the conditions in our case did not differ in any way in terms of instructions, and therefore a strategic adjustment of speed-accuracy trade-off is unlikely. We can speculate that presenting concerns (whether it be the participant's own or other's) diverts their attention a little bit from the task, and thereby leads them to perform the task more on auto-pilot, with lower accuracy and faster response times.

We have observed that self-reported thought “stickiness” has substantial effects on task performance in a laboratory study. Since task-unrelated thinking occupies between 10 and 50% of our daily thinking time (Antrobus et al., 1970; Smallwood et al., 2007; Killingsworth and Gilbert, 2010), and since maladaptive forms of task-unrelated thinking such as rumination have destructive consequences on productivity and well-being, studying when, how, and why this occurs is crucial. A previous experience sampling study of mind-wandering showed that when people reported their mind-wandering involved episodic memories, those were predominantly personal concerns (Song and Wang, 2012). Given our finding that self-reported stickiness affects performance in a very simple decision task, it would be interesting to include our stickiness question in experience sampling studies, and assess how it affects well-being in the real world. This question may be of particular interest when using experience sampling to chart the “thought trajectories” of depressed patients (van der Krieke et al., 2015).

While increases in self-reported stickiness may be related to tendencies for depression, it may also be interesting and relevant to examine the correlates and consequences of lower self-reported stickiness. For example, it has been shown that scores on the HINT, from which we took the question on mental stickiness, are negatively related to scores on a mindfulness questionnaire (Verplanken et al., 2007). Moreover, participants who scored high on the HINT showed a larger reduction in distress over disturbing pictures as a result of a mindfulness induction relative to participants who scored low on the HINT (Verplanken and Fisher, 2014). Mindfulness may help make people aware of their unconscious thought habits, and reduce the strength of those habits by consciously disengaging from these habits (Williams, 2010; Vago and Silbersweig, 2012). In particular, it has been suggested that mindfulness also reduces the tendency to get “stuck” in patterns of negative thinking (van Vugt et al., 2012). On the positive side, it is thought to cultivate a sense of “non-attachment” to worries and concerns (Sahdra et al., 2011), and equanimity to whatever occurs in experience (Desbordes et al., 2014). It would therefore be interesting to measure meditators' scores on mind-wandering tasks that include questions about the stickiness of their thinking.

In short, we have demonstrated that mind-wandering can vary in the amount of self-reported stickiness, and that this stickiness has consequences for task performance. As mental stickiness increases, the variance in response time does too. Given the close relatedness of mental stickiness to psychopathology, we think this thought probe question is an important tool in future studies of mind-wandering.

### Data sharing

The data, data analysis file in R markdown and the experiment code can be downloaded from http://www.ai.rug.nl/~mkvanvugt/SARTdataWeb.zip.

## Author contributions

MvV and NB conceived of the experiment. NB collected the behavioral data. MvV drafted the first version of the paper, all authors edited it and approved of the final version of the paper. All authors agree to be accountable for all aspects of the work.

## Funding

Marie Curie Career Integration grant (ACCDECMEM) to MvV, Seventh Framework Programme.

### Conflict of interest statement

The authors declare that the research was conducted in the absence of any commercial or financial relationships that could be construed as a potential conflict of interest.
